# The diagnostic challenge of dizziness: computed tomography and magnetic resonance imaging findings

**DOI:** 10.1590/0100-3984.2016.0054

**Published:** 2017

**Authors:** Bruno Niemeyer de Freitas Ribeiro, Rafael Santos Correia, Lívia de Oliveira Antunes, Tiago Medina Salata, Heraldo Belmont Rosas, Edson Marchiori

**Affiliations:** 1 Masters Student, MD, Neuroradiologist at the Instituto Estadual do Cérebro Paulo Niemeyer, Rio de Janeiro, RJ, Brazil.; 2 Full Member of the Colégio Brasileiro de Radiologia e Diagnóstico por Imagem (CBR), MD, Radiologist at the Hospital Universitário Walter Cantídio, Fortaleza, CE, Brazil.; 3 MD, Radiologist at the Hospital Casa de Portugal/3D Diagnóstico por Imagem, Rio de Janeiro, RJ, Brazil.; 4 Full Professor at the Universidade Federal do Rio de Janeiro (UFRJ), Rio de Janeiro, RJ, Brazil.

**Keywords:** Dizziness, Vertigo, Neuroimaging, Computed tomography, magnetic resonance imaging, Tontura, Vertigem, Neuroimagem, Tomografia computadorizada, Ressonância magnética

## Abstract

Dizziness is a prevalent symptom in the general population, accounting for a considerable share of physician office visits, and most causes are clinically treatable. It is also a common indication for neuroimaging studies, in order to identify a specific etiology and exclude surgical causes. Here, we illustrate the main peripheral and central causes of dizziness, discussing their possible differential diagnoses, as well as their most important image aspects.

## Abstract (pt)

Tontura é sintoma prevalente na população geral e responsável por uma parcela considerável de idas ao consultório médico, sendo a maior parte das causas tratáveis clinicamente. Além disso, é indicação frequente nos exames de neuroimagem, a fim de definir uma causa específica e excluir possíveis causas cirúrgicas. Neste ensaio ilustramos as principais causas periféricas e centrais de tontura, discutindo seus possíveis diagnósticos diferenciais, bem como seus aspectos de imagem mais relevantes.

## INTRODUCTION

The ability of human beings to remain upright, to accelerate, and to rotate, without wavering or falling, is called equilibrium, or balance. The maintenance of balance requires appropriate interaction among the vestibular, visual, and proprioceptive systems^(1)^. Disturbances in the relationship among these systems usually manifest as dizziness. Dizziness occurs in 5–10% of the world population and in 65% of individuals over 65 years of age^(1)^. The term is nonspecific and usually covers a range of presentations, the most common being vertigo (a false sensation of bodily movement), disequilibrium, and presyncope^(2)^. Vertigo is more often associated with disorders of the vestibular system and its connections, whereas disequilibrium is usually associated with neurological damage^(2)^, and it is not easy to make this distinction clinically.

## DISCUSSION

Various etiologies are associated with dizziness. Therefore, as part of the initial evaluation, cardiovascular, endocrine, pharmacological, and psychiatric causes need to be excluded before imaging studies are considered.

In this study, we will discuss imaging findings related to dizziness. We have organized those findings by etiologic class, including neoplastic, infectious/inflammatory, anatomical, traumatic/postoperative, and other causes.

### Neoplastic causes

#### Meningioma

**Figure 1:**
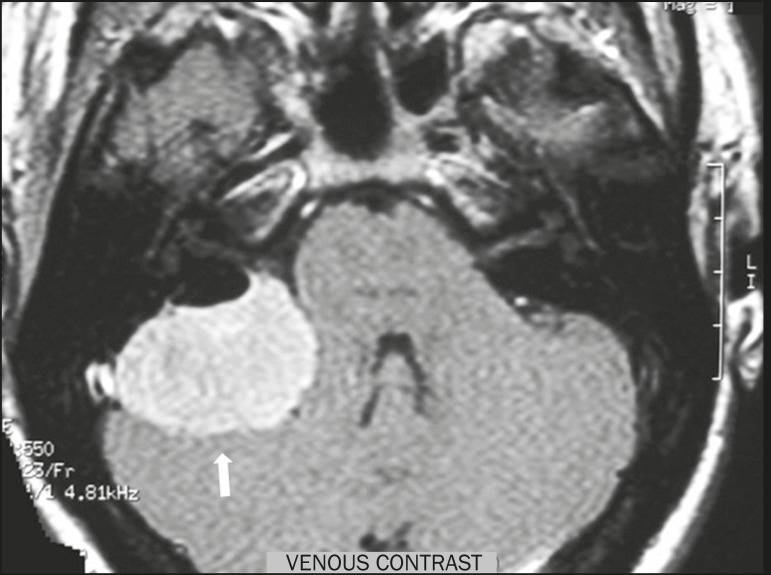
Meningioma. Contrast-enhanced, axial T1-weighted MRI sequence, showing a homogeneous meningioma (arrow), with intense enhancement, located in the posterior fossa and encroaching upon the adjacent cerebellar parenchyma.

Meningioma is the most common extra-axial tumor in adults and the second most common lesion in the cerebellopontine angle. Meningiomas usually have a homogeneous appearance on computed tomography (CT) and magnetic resonance imaging (MRI), with intense contrast enhancement (Figure 1). The presence of the dural tail sign is suggestive of, although not specific for, the diagnosis. Despite slow growth, when located in the posterior fossa, meningiomas can have a compressive effect on the cerebellum, consequently causing dizziness^(3)^.

#### Schwannoma

**Figure 2:**
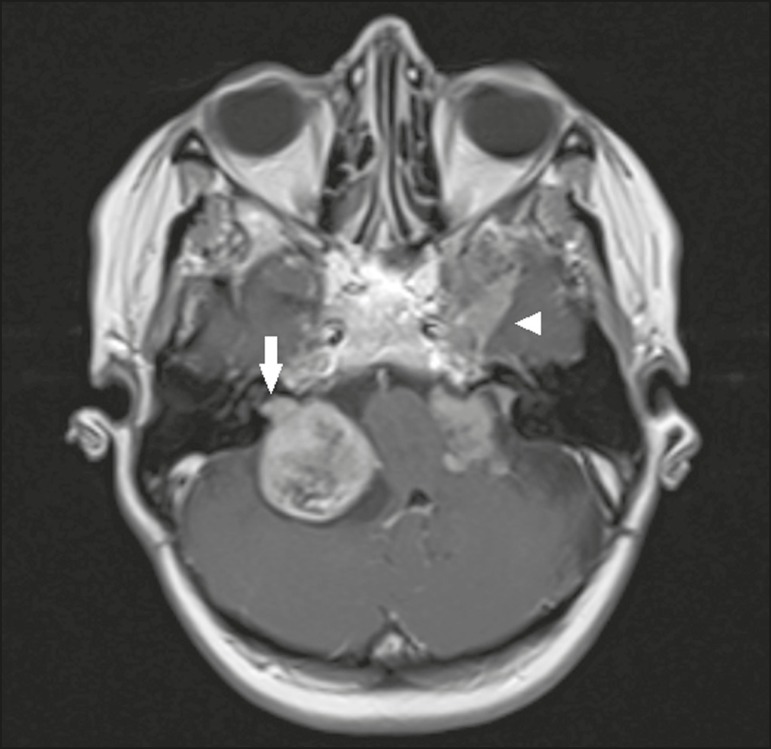
Schwannoma. Contrast-enhanced axial T1-weighted MRI sequence. Patient with type 2 neurofibromatosis presenting bilateral (right) schwannoma, extending to the internal auditory canal (arrow), with a meningioma (arrowhead) visible in the left middle fossa.

Schwannoma is the most common lesion in the cerebellopontine angle, usually (when small) has a homogeneous appearance, and presents the Antoni A type of histological pattern. Insinuation into and enlargement of the internal auditory canal, as depicted in Figure 2, is suggestive of, although not specific for, the diagnosis. The main nerve involved is the eighth cranial nerve, manifesting mainly with tinnitus and hearing loss, although schwannomas can cause dizziness when they exert a compressive effect on the cerebellum^(3)^.

#### Hemangioblastoma

**Figure 3:**
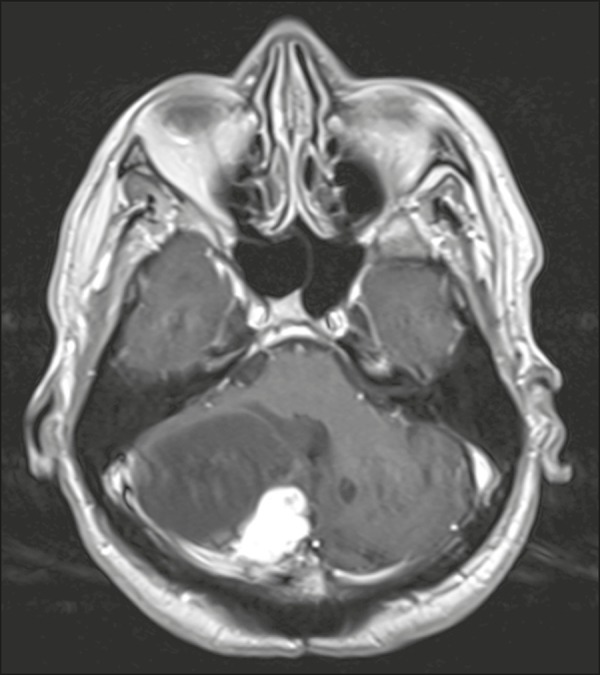
Hemangioblastoma. Contrast-enhanced axial T1-weighted MRI sequence showing a solid-cystic lesion, with intense enhancement of the solid portion, affecting the right cerebellar hemisphere.

Hemangioblastomas are more common in the cerebellum, are associated with von Hippel–Lindau disease, and can be accompanied by polycythemia, because they are capable of producing erythropoietin^(4)^. They are often cystic tumors with a solid mural component; purely solid Hemangioblastomas, which have a higher rate of local recurrence, occur in 30% of cases^(4)^. They are lesions with high contrast uptake, and, on MRI, the solid component shows an isointense signal on T1-weighted images and a hyperintense signal on T2-weighted images, sometimes being accompanied by facilitated diffusion, as shown in Figure 3^(4,5)^.

#### Glomus jugulare and glomus jugulotympanicum tumors

**Figure 4:**
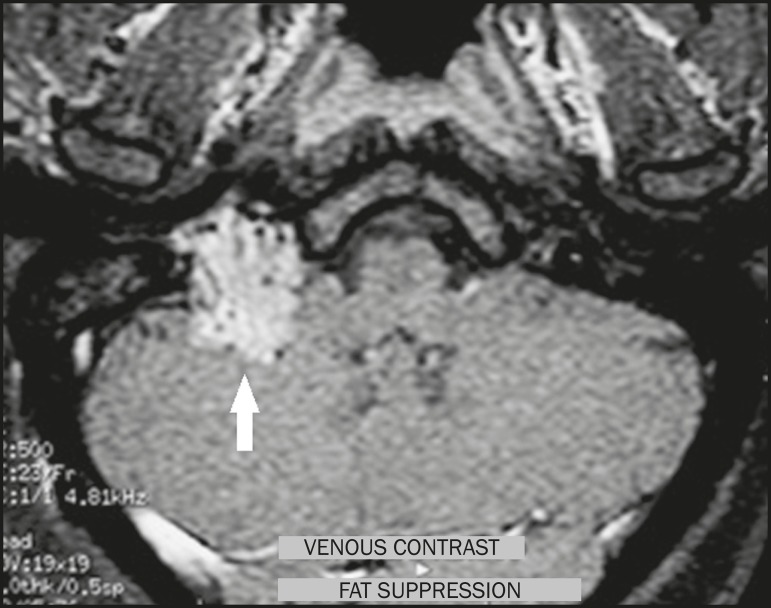
Glomus tumor. Contrast-enhanced axial T1-weighted MRI sequence showing a lesion with marked enhancement (arrow), containing areas consistent with flow voids.

Glomus jugulare and glomus jugulotympanicum, tumors of the chemoreceptor system, are the main primary tumors of the jugular foramen. The majority of such tumors are benign, they present aggressive behavior. On CT, they manifest as irregular bone destruction with significant contrast enhancement. On MRI, they present low signal intensity on T1-weighted images and high signal intensity on T2-weighted images, also with significant contrast enhancement. Larger glomera can present internal flow voids^(6)^, as depicted in Figure 4.

#### Endolymphatic sac tumor

**Figure 5:**
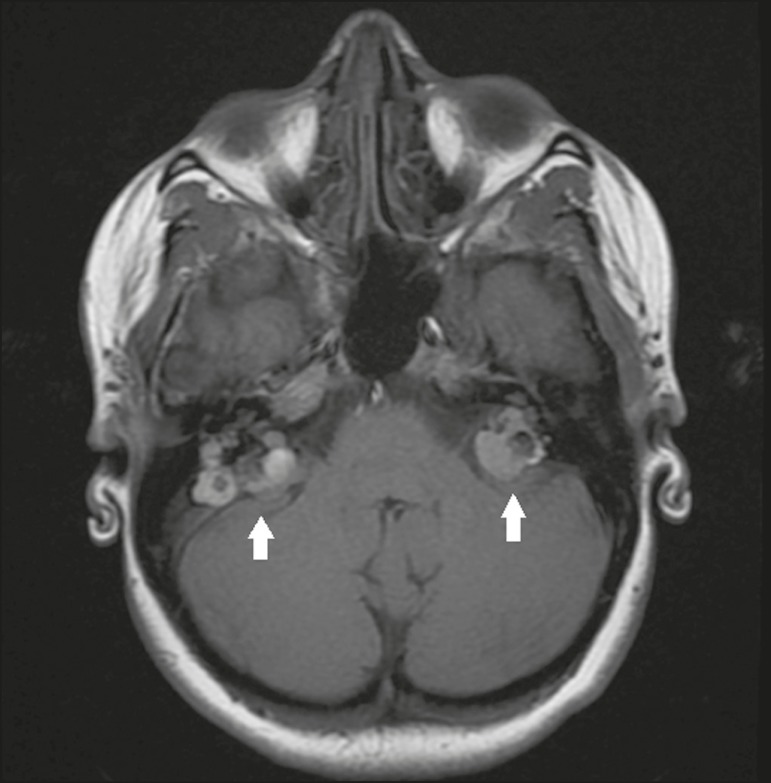
Endolymphatic sac tumor. Non-contrast-enhanced axial T1-weighted MRI sequence. Patient with von-Hippel-Lindau disease presenting a bilateral endolymphatic sac tumor (arrows).

Endolymphatic sac tumors are rare tumors of the posterior region of the petrous portion of the temporal bone that are slow growing and occur sporadically in most cases. Although they are not malignant, they are locally invasive. In 15% of cases, endolymphatic sac tumors are associated with von Hippel-Lindau disease^(4)^. On CT, the bone destruction is either geographic or has a moth-eaten appearance, with a peripheral rim of calcification. On MRI, the signal is heterogeneous, with hyperintense foci seen within the lesion in a T1-weighted sequence (Figure 5).

#### Metastasis

**Figure 6:**
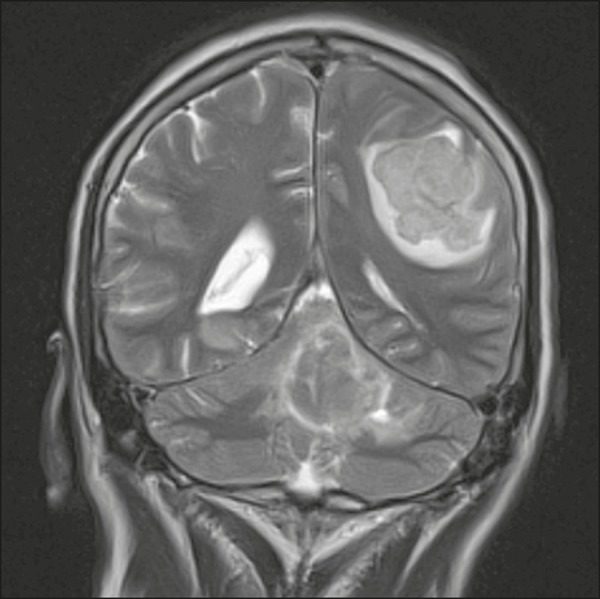
Metastasis. Coronal T2-weighted MRI sequence. Patient with breast neoplasm presenting two lesions with perilesional edema.

Metastases are the principal malignant neoplasm involving the brain and are more common in the supratentorial compartment because of its greater vascularization. When they occur in the infratentorial compartment, they often provoke dizziness. The most common primary sites are the breast, lung, kidney, stomach, and prostate. There are no specific imaging features, making it difficult to differentiate metastases from other lesions. Metastases should be considered in patients with known primary neoplasia or multiple brain lesions (Figure 6).

### Infectious/inflammatory causes

#### Otomastoiditis

**Figure 7:**
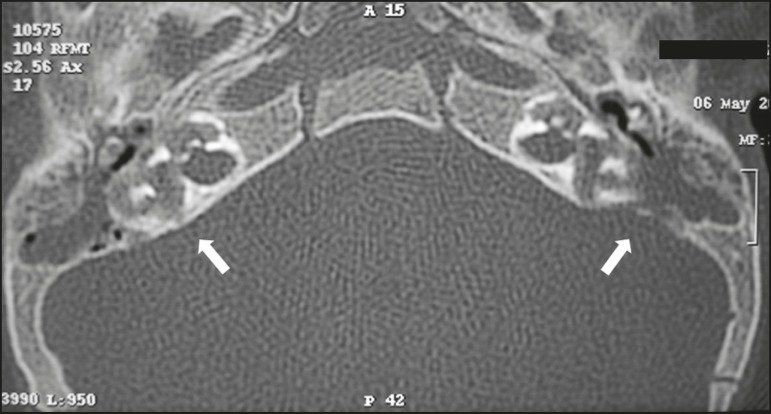
Otomastoiditis in an immunocompromised patient. Axial CT, with a bone window, showing bilateral otomastoiditis (arrows), accompanied by pronounced bone erosion, extending to the labyrinth, in a patient with high fever and pain on palpation of the mastoid region, who developed dizziness.

**Figure 8:**
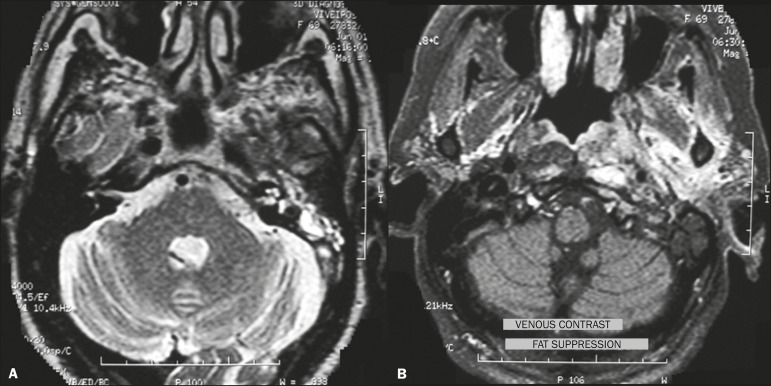
*Pseudomonas aeruginosa* otomastoiditis in a patient with poor glycemic control. A: Axial T2-weighted MRI sequence showing left-sided otomastoiditis with marked involvement of the ipsilateral labyrinth. B: Contrast-enhanced axial T1-weighted MRI sequence of the same patient. Note the extensive involvement, even reaching the masticator space.

**Figure 9:**
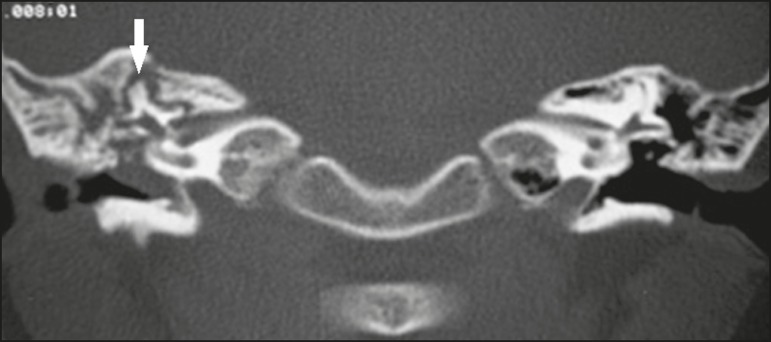
Osteomyelitis-complicated otomastoiditis. Coronal CT, with a bone window, showing bone sequestration (arrow) within right-sided otomastoiditis.

**Figure 10:**
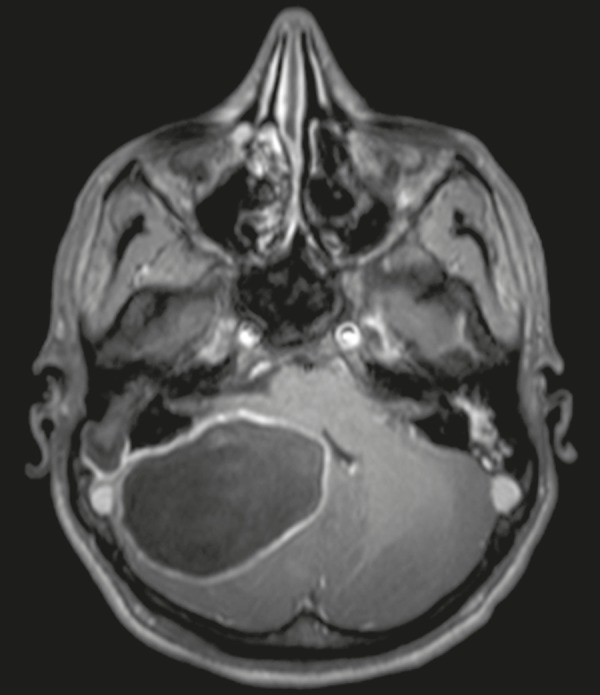
Cerebellar abscess-complicated otomastoiditis. Gadolinium-contrast-enhanced axial T1-weighted MRI sequence showing a lesion with enhancement of its walls, involving the cerebellum.

*Otomastoiditis involves infection of the tympanic and mastoid cavities, typically caused by bacterial agents, the most common being Streptococcus pneumoniae* and *Haemophilus influenzae*. Immunocompromised patients present risk factors for uncommon infectious agents, as well as being prone to more extensive, rapidly progressive impairment^(7)^, which can be detected on CT and MRI (Figures 7 and 8, respectively). On CT, uncomplicated otomastoiditis commonly presents as material with a hypointense signal, without bone erosion. On MRI, no restricted diffusion is expected. When not treated properly, it can progress to osteomyelitis (Figure 9) or intracranial complications, including meningitis, abscesses (Figure 10), and venous thrombosis. The incidence of those complications has declined substantially because of the widespread use of antibiotics.

#### Cholesteatoma

**Figure 11:**
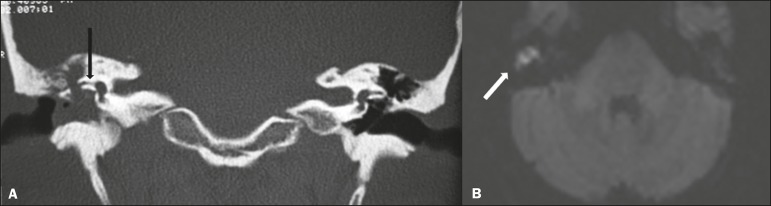
Cholesteatoma. A: Coronal CT, with a bone window, showing material with soft tissue density affecting the right tympanic cavity and causing intense erosion of the bone structures, including labyrinthine fistula with lateral semicircular canal (arrow). B: Axial diffusion-weighted MRI sequence showing a right-sided cholesteatoma (arrow) with a hyperintense signal.

Cholesteatoma involves proliferation of keratinized stratified squamous epithelium, with pathological characteristics identical to those of epidermoid cyst. It can be acquired or congenital, occurring in the pars flaccida or pars tensa. In most cases, it is acquired and occurs in the pars flaccida. On CT, cholesteatomas typically appear as lesions with soft-tissue density in Prussak’s space, accompanied by erosion of the ossicular chain and lateral wall of the attic (epitympanic recess), and can also be accompanied by labyrinthine fistulas. In functional diffusion-weighted MRI sequences, cholesteatomas show high signal intensity, facilitating the distinction with inflammatory granulation tissue (Figure 11).

#### Acute cerebellitis

**Figure 12:**
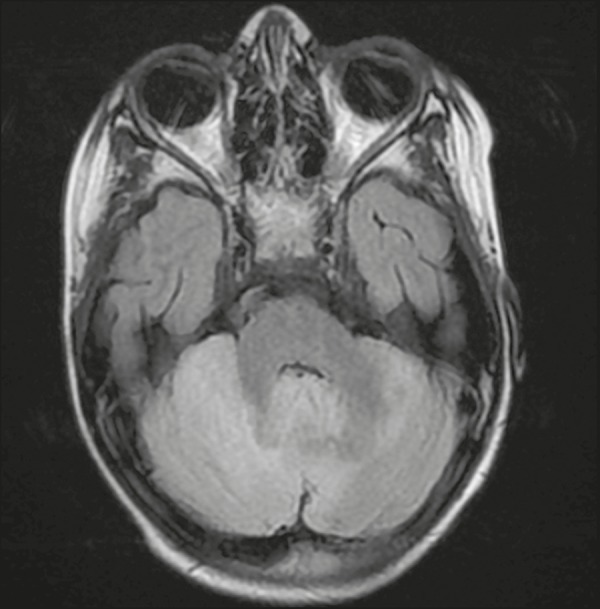
Cerebellitis caused by infection with the Epstein-Barr virus. Axial slice in a fluid-attenuated inversion recovery sequence, showing diffuse hyperintensity in the cerebellum, especially in the right hemisphere.

Most common in children, acute cerebellitis is encephalitis that is restricted to the cerebellum, affecting one or both hemispheres (Figure 12). Although the varicella-zoster virus is the leading cause of acute cerebellitis, other viral agents, such as echovirus and poliovirus, have been implicated. There have also been reports of bacterial causes.

#### Infectious involvement of cranial nerves

**Figure 13:**
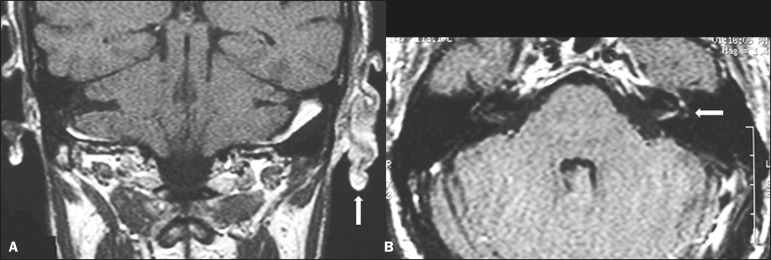
Ramsay Hunt syndrome. A: Coronal T1-weighted MRI sequence showing enlargement of the soft tissues within the left auricular cartilage (arrow). B: Contrast-enhanced axial T1-weighted MRI sequence showing enhancement of the vestibulocochlear and facial nerves on the left, as well as discrete enhancement of the ipsilateral labyrinth (arrow).

Ramsay Hunt syndrome typically corresponds to reactivation of a latent focus of varicella-zoster virus infection in the geniculate ganglion, being characterized clinically by intense earache, erythematous vesicular rash, peripheral facial paralysis, and dizziness^(8)^. MRI scans can show enlargement of the soft parts of the auricle, together with contrast enhancement of the facial and vestibulocochlear nerves^(8)^, as depicted in Figure 13.

### Anatomical causes

#### Superior semicircular canal dehiscence

**Figure 14:**
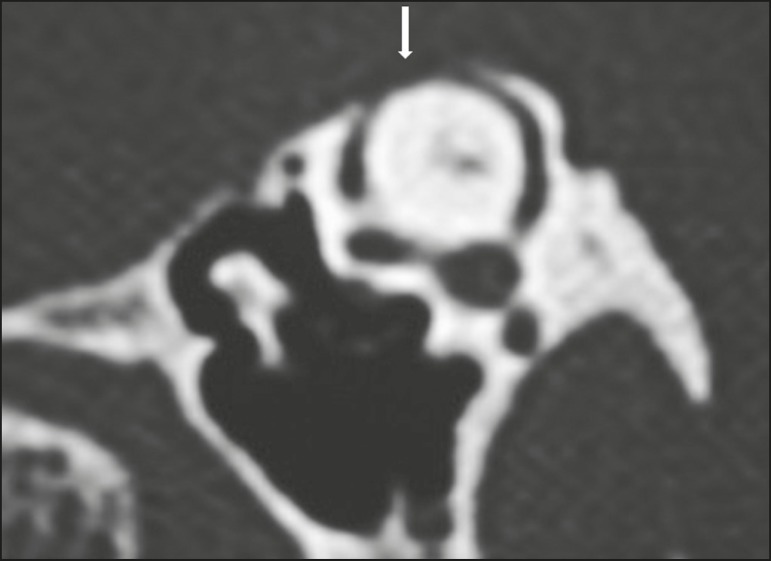
Superior semicircular canal dehiscence. CT with a bone window and oblique reconstruction in the Pöschl plane, showing a bone defect in the superior semicircular canal.

Superior semicircular canal dehiscence consists of the absence of the bone layer that covers the canal and can be accompanied by vestibular symptoms induced by intense sound stimuli or modification of intracranial pressure or of the pressure in the middle ear, with a prevalence of 0.7% in the general population^(9)^. Not all individuals with superior semicircular canal dehiscence are symptomatic. On CT with a bone window and oblique reconstruction in the Pöschl plane, a bone defect can be seen in the superior semicircular canal (Figure 14).

#### Other anatomical changes

**Figure 15:**
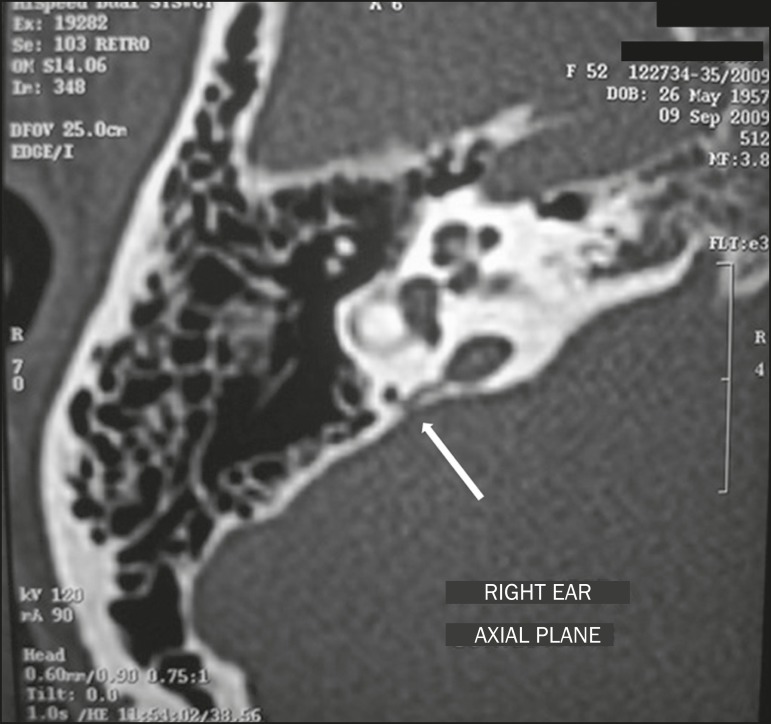
Congenital perilymph fistula. Axial CT, with a bone window, showing anomalous communication between the vestibular aqueduct and the posterior semicircular canal (arrow).

Anatomical changes such as jugular bulb diverticulum, an aberrant carotid artery in the middle ear, and congenital perilymph fistula can cause dizziness (Figure 15). In general, external ear malformations are accompanied by malformations of the middle ear, because they have the same embryological origin, malformations of the inner ear coexisting with those of the external ear in only 15–20% of cases^(10)^.

### Causes related to trauma or postoperative complications

#### Fractures

**Figure 16:**
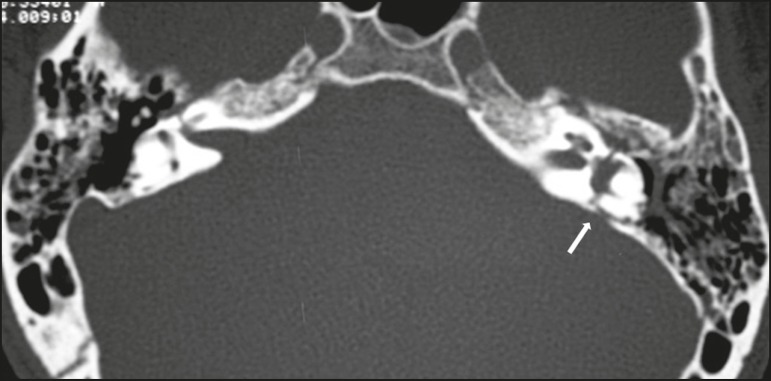
Fracture. Axial CT, with a bone window, showing a transverse fracture line on the left side (arrow), with translabyrinthine involvement.

Most skull fractures result from high-energy trauma. The traditional classification indicates that there is a relationship between the fracture line and the longest axis of the petrous portion of the temporal bone, temporal bone fractures being categorized as longitudinal, transverse, or mixed. The longitudinal type typically occurs in temporoparietal trauma, mainly affecting the extra-labyrinthine portion, the main complications being ossicular lesion and hemotympanum. Transverse temporal bone fractures usually occur in fronto-occipital traumas, are more often associated with dizziness, due to translabyrinthine involvement, and can result in damage to the facial nerve (Figure 16).

#### Remote cerebellar hemorrhage

**Figure 17:**
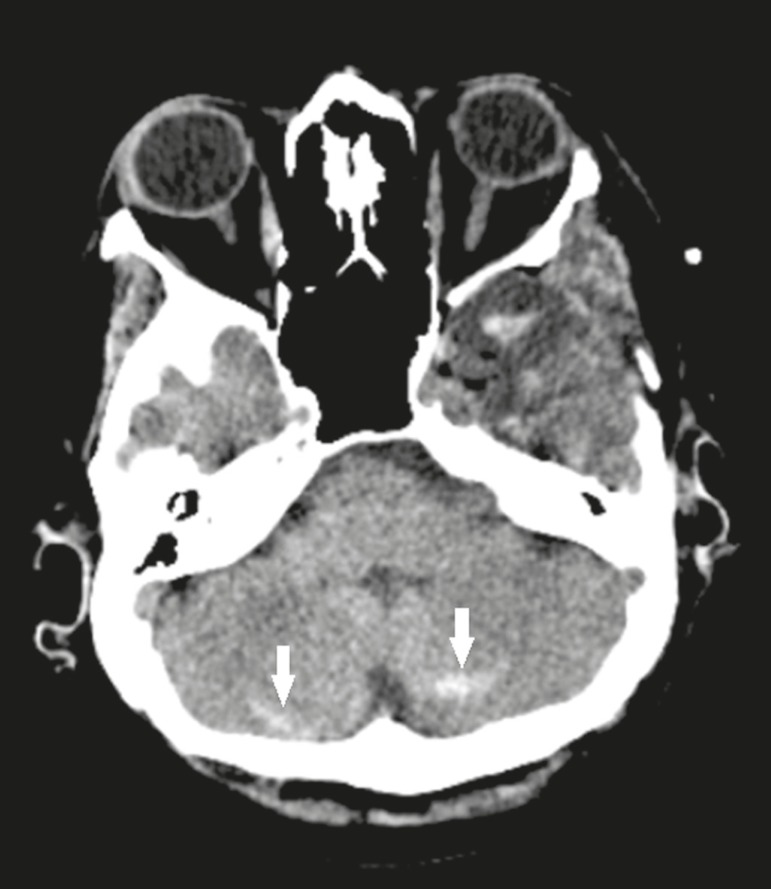
Remote cerebellar hemorrhage. Axial CT, with a parenchymal window, showing dense foci with a striped aspect, affecting both cerebellar hemispheres (arrows), consistent with bleeding, in a patient recently submitted to neurosurgery.

In most cases, remote cerebellar hemorrhage is a benign, self-limiting entity. It is commonly related to supratentorial neurosurgery and can be asymptomatic. CT shows dense foci with a striped aspect affecting one or both cerebellar hemispheres, consistent with bleeding, as shown in a patient with a recent history of neurosurgery in Figure 17. In susceptibility-weighted imaging sequences, foci of signal loss can be seen.

### Other causes

**Figure 18:**
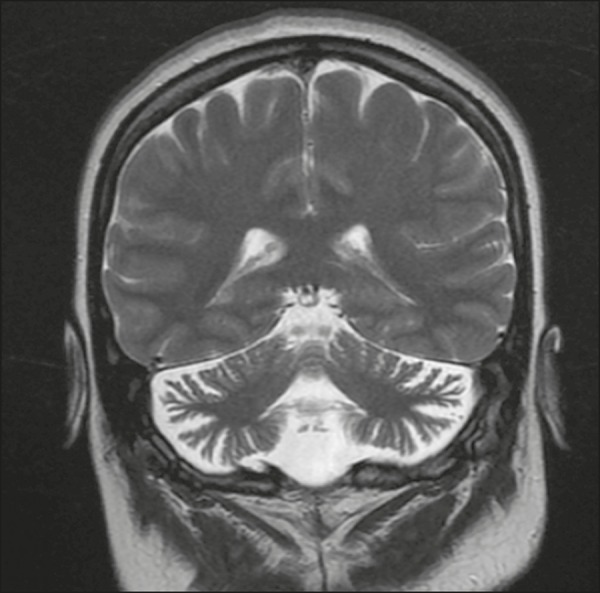
Cerebellar atrophy in a chronic phenytoin user. Coronal T2-weighted MRI sequence showing marked atrophy of the cerebellum, disproportionate to that observed in the rest of the brain parenchyma, in an 18-year-old patient who had been treated with phenytoin since the age of 5 years.

Chronic users of phenytoin or individuals with acute phenytoin intoxication can develop cerebellar atrophy, which produces permanent cerebellar lesion, with atrophy of the cerebellar vermis and cerebellar hemispheres (Figure 18). There is some controversy as to whether phenytoin use alone is responsible for cerebellar atrophy, given that it can also be caused by hypoxia due to convulsive seizures. However, Rapport et al.^(11)^ reported cerebellar atrophy in a patient treated prophylactically with phenytoin, and that patient had no history of epileptic seizures. In patients with a clinical history consistent with a diagnosis of cerebellar atrophy, MRI shows marked atrophy of the cerebellum, disproportionate to that observed in the rest of the brain parenchyma.

## CONCLUSION

Imaging is a quite useful tool in the context of patients with dizziness, because it is capable of providing additional information that is fundamental to the diagnosis, therapeutic planning, and follow-up. The radiologist must be alert to its differential diagnoses, in order to inform the clinical decision-making process.
